# Efficacy and safety of tildrakizumab 100 mg for plaque psoriasis in patients randomized to treatment continuation vs treatment withdrawal with retreatment upon relapse in reSURFACE 1

**DOI:** 10.1111/jdv.17124

**Published:** 2021-02-12

**Authors:** W. Cantrell, P. Lee, A.M. Mendelsohn, S.J. Rozzo, W. Liao

**Affiliations:** ^1^ Village Dermatology Birmingham AL USA; ^2^ Center for Clinical Studies Webster TX USA; ^3^ Sun Pharmaceutical Industries, Inc. Princeton NJ USA; ^4^ Department of Dermatology University of California San Francisco San Francisco CA USA

Editor

Chronic moderate‐to‐severe plaque psoriasis frequently requires long‐term biologic treatment, but treatment gaps resulting in relapse are not uncommon.^1^, ^2^, ^3^, ^4^ Biologic efficacy can be recaptured during subsequent retreatment but typically at a lower rate, or to a lesser extent, than the response achieved during initial treatment.^5^, ^6^, ^7^

Tildrakizumab is a high‐affinity, humanized, immunoglobulin G1κ, anti–interleukin‐23p19 monoclonal antibody approved to treat plaque psoriasis.^8^, ^9^, ^10^ The 64‐week phase 3 reSURFACE 1 (NCT01722331) study in adult patients with moderate‐to‐severe plaque psoriasis required tildrakizumab‐treated patients who achieved ≥75% improvement from baseline Psoriasis Area and Severity Index score (PASI 75; responders) at Week 28 to be rerandomized to either continued tildrakizumab treatment or withdrawal (placebo) with retreatment with their initial dose of tildrakizumab upon relapse (loss of 50% of maximum PASI benefit from baseline).^8^ This *post hoc* analysis evaluated residual disease in tildrakizumab 100 mg responders in reSURFACE 1 who were continuously treated and those rerandomized to treatment interruption and retreatment upon relapse. Time to response and extent of response after retreatment were also evaluated. Data were summarized using descriptive statistics. Missing data were imputed by last observation carried forward.

Disease activity (as median absolute PASI score) assessed at baseline, Week 28, Week 52 and Week 64 is shown in Table 1. Of 116 patients continuously treated with tildrakizumab 100 mg after Week 28, 92.6%, 81.5% and 49.6% achieved PASI 50, PASI 75 and PASI 90, respectively, at Week 64 (Fig. 1). Complete clearance was achieved by 27.4% of patients at Week 64. At Week 64, the median [interquartile range (IQR)] percentage improvement (decrease) from baseline PASI score was 92.4% (84.4%, 100.0%).

**Table 1:** Median absolute PASI scores by treatment condition and time point

	Baseline	Week 28	Week 52	Week 64
Continuous TIL 100 mg (*n* = 108)	19.7 (14.2, 23.0)	1.0 (0.0, 2.2)	1.0 (0.0, 2.4)	1.2 (0.0, 3.0)
TIL → PBO, no relapse (*n* = 52)	18.6 (14.4, 21.6)	0.8 (0.0, 3.2)	2.6 (0.8, 5.2)	4.0 (2.0, 7.4)

	Baseline	Week 28	At time of relapse	Week 12 after retreatment
TIL → PBO, relapse (*n* = 61)	20.3 (14.3, 22.9)	0.8 (0.0, 2.2)	11.0 (8.6, 16.2)	2.7 (0.8, 4.6)

Numbers are median (IQR). IQR, interquartile range; PASI, Psoriasis Area and Severity Index; PBO, placebo; TIL, tildrakizumab.

**Figure 1:**
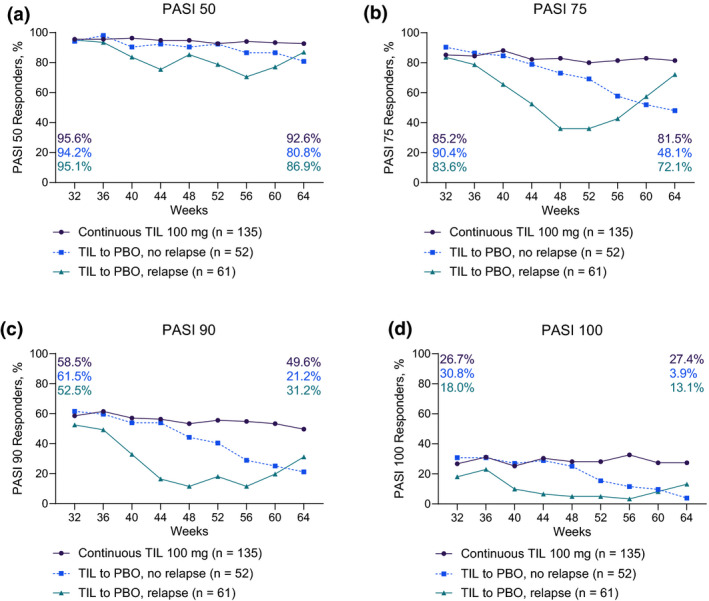
Rates of (a) PASI 50; (b) PASI 75; (c) PASI 90; and (d) PASI 100 responders over time in patients who received continuous tildrakizumab 100 mg in part 3 of reSURFACE 1, patients who were rerandomized to placebo and did not relapse, or patients who were rerandomized to placebo and relapsed. PASI, Psoriasis Area and Severity Index; PBO, placebo; TIL, tildrakizumab.

Of 113 patients rerandomized to placebo at Week 28 (last tildrakizumab dose at week 16), 52 (46%) did not relapse for 48 weeks following their last dose of tildrakizumab. Of these, the proportions achieving PASI 50, PASI 75 and PASI 90 at Week 64 were 80.8%, 48.1% and 21.2%, respectively (Fig. 1). Complete clearance was achieved by 3.9% of these patients at Week 64. Their median (IQR) percentage improvement from baseline PASI score at Week 64 was 75.7% (57.8%, 88.8%).

There were 61 (54.0%) patients rerandomized to placebo at Week 28 who relapsed by Week 64 and were retreated with tildrakizumab 100 mg. Median (IQR) time to relapse was 238 (167, 294) days. Among 51 patients with ≥12 weeks of retreatment data, median (IQR) time to regain PASI 75 response was 28 (28, 48) days; response was regained by 49 (96.1%) in <12 weeks of retreatment. Of patients who relapsed and were retreated, the proportion of PASI 50, PASI 75 and PASI 90 responders was, respectively, 86.9%, 72.1% and 31.2% at Week 64 (Fig. 1). Complete clearance was achieved by 13.1% at Week 64 (Fig. 1). Median PASI at time of loss of response was 11.0 (8.6, 16.2).

Of Week 28 responders, 112/116 (96.6%) who continued to receive tildrakizumab 100 mg and 104/113 (91.2%) rerandomized to placebo completed Week 64. No patient experienced disease rebound (>125% worsening from baseline PASI score). Prespecified adverse events of special interest^8^ occurred in <3% of patients, with no adverse events in patients receiving either placebo or tildrakizumab 100 mg who relapsed after rerandomization.

From Weeks 28–64 of reSURFACE 1, tildrakizumab 100 mg was well tolerated and efficacious in patients receiving continuous treatment; patients withdrawn to placebo recovered response within a median of 28 days. The durability of tildrakizumab responses and rapid regain of efficacy after relapse and retreatment support long‐term clinical use of tildrakizumab for the treatment of moderate‐to‐severe psoriasis.

## Conflicts of interest

WC has no disclosures on file. PL has served as an investigator for Merck. AMM is an employee of Sun Pharmaceutical Industries, Inc.; and has individual shares in Johnson and Johnson, and as part of retirement account/mutual funds. SJR is an employee of Sun Pharmaceutical Industries, Inc. WL has conducted research funded by AbbVie, Amgen, Janssen, Leo, Novartis, Pfizer, Regeneron/Sanofi and TRex Bio.

## Funding sources

These studies were funded by Merck Sharp & Dohme Corp., a subsidiary of Merck & Co., Inc., Kenilworth, NJ, USA. Analyses were funded by Sun Pharmaceutical Industries, Inc., Princeton, NJ, USA. Medical writing support was provided by Atreju Lackey, PhD, of AlphaBioCom, LLC, and funded by Sun Pharmaceutical Industries, Inc.
